# Assessment of Airway Distensibility by the Forced Oscillation Technique: Reproducible and Potentially Simplifiable

**DOI:** 10.3389/fphys.2017.00223

**Published:** 2017-04-12

**Authors:** Samuel Mailhot-Larouche, Mélanie Lachance, Michela Bullone, Cyndi Henry, Ronald J. Dandurand, Louis-Philippe Boulet, Michel Laviolette, Gregory G. King, Claude S. Farah, Ynuk Bossé

**Affiliations:** ^1^Department of Medicine, Quebec Heart and Lung Institute, Université LavalQuebec, QC, Canada; ^2^Department of Veterinary Science, University of TurinTurin, Italy; ^3^Meakins-Christie Laboratories, FOT Unit, Centre for Innovative Medicine, Montreal Chest Institute, McGill University Health Centre and McGill UniversityMontreal, QC, Canada; ^4^Woolcock Institute of Medical ResearchSydney, NSW, Australia; ^5^Sydney Medical School, University of SydneySydney, NSW, Australia; ^6^Department of Respiratory Medicine, Royal North Shore HospitalSydney, NSW, Australia; ^7^Department of Respiratory Medicine, Concord HospitalSydney, NSW, Australia; ^8^Department of Clinical Medicine, Faculty of Medicine and Health Sciences, Macquarie UniversitySydney, NSW, Australia

**Keywords:** forced oscillation technique, respiratory system conductance, remodeling, airway caliber, lung volume, breathing maneuvers

## Abstract

A non-invasive index of airway distensibility is required to track airway remodeling over time. The forced oscillation technique (FOT) provides such an index by measuring the change in respiratory system conductance at 5 Hz over the corresponding change in lung volume (ΔGrs_5_/ΔV_L_). To become useful clinically, this method has to be reproducible and easy to perform. The series of breathing maneuvers required to measure distensibility would be greatly facilitated if the difficulty of breathing below functional residual capacity (FRC) could be precluded and the number of maneuvers could be reduced. The distensibility at lung volumes below FRC is also reduced by several confounders, suggesting that excluding data points below FRC should provide a better surrogate for airway remodeling. The objectives of this study were to investigate the reproducibility of airway distensibility measured by FOT and to assess whether the method could be simplified to increase feasibility. Distensibility was measured at three separate occasions in 13 healthy volunteers. At each visit, three deflationary maneuvers were performed, each consisting of tidal breathing from total lung capacity (TLC) to residual volume by slowly decreasing the end-expiratory volume on each subsequent breath. Distensibility was calculated by using either all data points from TLC to residual volume (RV) or only data points from TLC to FRC for either all three or only the first two deflationary maneuvers. Intra-class correlation coefficients (ICC) were used to assess reproducibility and Bland-Altman analyses were used to assess the level of agreement between the differently calculated values of distensibility. The results indicate that distensibility calculated using all data points is reproducible (ICC = 0.64). Using data points from TLC to FRC slightly improved reproducibility (ICC = 0.68) and increased distensibility by 19.4%, which was expected as distensibility above FRC should not be affected by confounders. Using only data points within the first two maneuvers did not affect reproducibility when tested between TLC and FRC (ICC = 0.66). We conclude that a valuable measure of airway distensibility could potentially be obtained with only two deflationary maneuvers that do not require breathing below FRC. This simplified method would increase feasibility without compromising reproducibility.

## Introduction

Remodeling of the airway wall is an important feature of many respiratory diseases (Hirota and Martin, 2013). Unfortunately, treatments directed specifically toward reversing airway remodeling are currently non-existent (Hirota and Martin, 2013). In addition, the effect of mainstay therapies for respiratory diseases on airway remodeling is not clear (Durrani et al., 2011). A major problem that impedes the progress in that research area is the lack of a non-invasive measure to assess prospectively the changes in remodeling that occur during the natural course of disease development and during treatment (Prakash et al., 2017). The current gold standard to assess remodeling is the histological evaluation of bronchial biopsies, which is impractical for obvious reasons. The measurement of airway distensibility by non-invasive physiological methods may offer mechanistically and clinically useful information related to remodeling.

Airway distensibility is defined as the change in airway caliber over a change in either lung volume or airway distending pressure. It is a measure of the ease and the extent by which the airways dilate in response to changes in distending stress. Brown and coworkers pioneered a method to assess airway distensibility non-invasively in human subjects using the forced oscillation technique (FOT) (Brown et al., 2004). FOT measures the respiratory system conductance (Grs) in real-time by the application of forced oscillations at specified frequencies in the subject's mouth while airflow and pressure are measured continuously at the mouth. To measure airway distensibility, the subject is instructed to take a deep inspiration to total lung capacity (TLC) and then to breathe tidally by slowly decreasing the end-expiratory volume down to residual volume (RV) (Kelly et al., 2012). This maneuver is hereafter called a “deflationary maneuver” and is usually repeated three times to obtain a sufficient number of data points. It is understood that Grs at 5 Hz (Grs_5_) measured at times of zero flow (at the end of each inspiration and expiration) mainly reflects the caliber of the airways. By plotting all the values of Grs_5_ at zero flow vs. their corresponding lung volume, airway distensibility (ΔGrs_5_/ΔV_L_) can be calculated at any chosen lung volume (Kelly et al., 2012).

Airway distensibility measured by FOT is reduced in patients with asthma (Brown et al., 2007) and chronic obstructive pulmonary disease (Baldi et al., 2010) compared with healthy individuals. Airway distensibility in asthmatic subjects also improves after a 12-week period of inhaled corticosteroid treatment (Kermode et al., 2011). This suggests that the method might be suitable to track changes caused by a pharmacological treatment. Yet, the current method has not been tested adequately for reproducibility. To be clinically useful in monitoring the changes in airway distensibility over time, and potentially as a surrogate for airway remodeling, this method has to provide reproducible results when tested repeatedly on the same individuals. Furthermore, a simpler series of breathing maneuvers would make the measurement of airway distensibility by FOT more feasible and more applicable in the clinical arena.

The first aim of this study was to assess the reproducibility of airway distensibility measured by FOT in healthy subjects. As exploratory aims, we then reanalysed the data to seek whether reducing lung volume excursions from TLC to FRC, rather than to RV, and whether reducing the number of deflationary maneuvers from three to two would affect the values of airway distensibility and its reproducibility.

## Methods

### Subjects

Thirteen healthy subjects were enrolled in this study from August 2015 to June 2016. All subjects had no history of respiratory disease and 12/13 were lifetime non-smokers. One subject was a current smoker. The Ethics Committee of the Quebec Heart and Lung Institute (QHLI) approved the study and all subjects gave written informed consent.

### Study design

**Figure 1:**
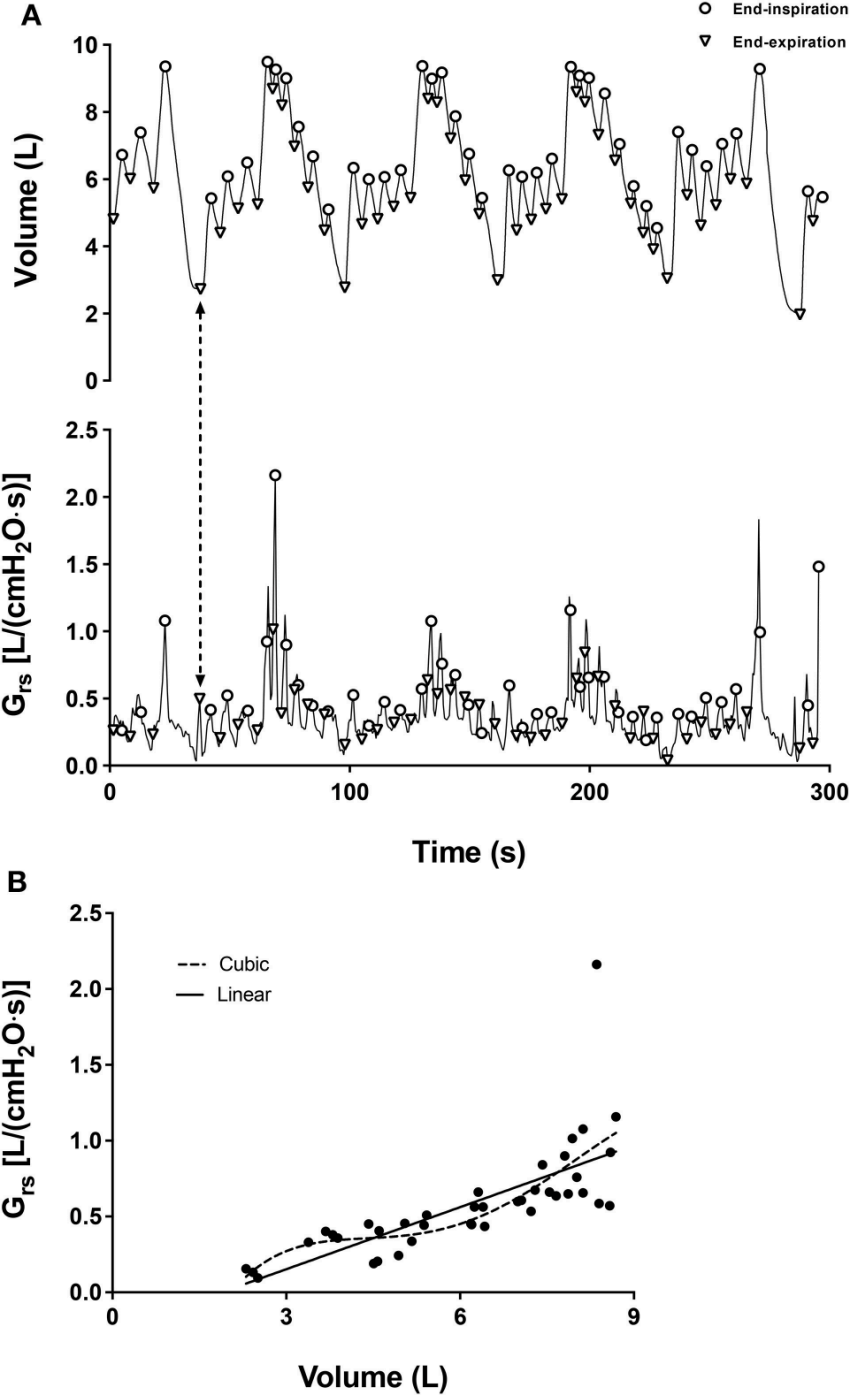
**(A)** Representative tracings of volume and Grs_5_ for one subject in one visit are displayed in the upper and lower panels, respectively. The triangles and the circles in the upper panel depict the lung volumes at end-expiration and end-inspiration, respectively. Triangles or circles on the Grs_5_ trace that are aligned vertically to the same symbols on the volume trace are Grs_5_ at time-points corresponding to end-expiratory or end-inspiratory volumes, respectively (e.g., the dashed line with double-ended arrows indicates the lung volume and the corresponding Grs_5_ at an end-expiratory time-point). **(B)** A plot showing the relationship between Grs_5_ at zero flow and lung volume. Each solid circle relates the simultaneous readouts of lung volume and Grs_5_ at a time-point corresponding to either end-expiration or end-inspiration. The dashed line is the curve of a cubic equation that best fitted the data. The curve derivatives represent airway distensibility at any chosen lung volume. The solid line is a linear regression that best fitted the data. The slope of that line is airway distensibility across the chosen range of lung volumes (between residual volume and total lung capacity in this example).

Airway distensibility was measured on three different occasions at least 24 h apart. Conventional spirometry was performed at the beginning of each visit. The first visit also included determination of lung volumes in a body plethysmograph (Platinum Elite™ body plethysmograph with RTD, MGC Diagnostics Corporation, Saint Paul, MN). Airway distensibility was then measured once at each visit by an FOT device (TremoFlo, Thorasys, Montreal, QC) using the series of breathing maneuvers described next, without coming off the mouthpiece (Kelly et al., 2012). First, a slow inhalation was taken to TLC followed by a slow expiration to RV. Next, subjects inhaled to TLC and then breathed at near tidal volumes with the aim of slowly decreasing end-expiratory lung volumes on each subsequent breath until RV was reached. The latter is called the “deflationary maneuver.” The subject then breathed at tidal volume for a few breaths in order to recover. The deflationary maneuver was repeated twice with recovery breathing at tidal volume after each of them. Finally, a slow inhalation to TLC followed by a slow exhalation to RV terminated the protocol (Figure 1A). Slow maneuvers were performed because the device cannot measure flow rate exceeding 2 liters per second. The subjects were instructed to control inflations and lung volumes without closing the glottis; i.e., to control breathing using respiratory muscles only.

The values of respiratory system conductance at 5 Hz (Grs_5_) at the end of each inspiration and expiration, when flow is zero (Figure 1A), were then plotted to their corresponding lung volumes (Figure 1B). The change in lung volume was calculated from the integration of flow. By ensuring that maximal lung inflation was achieved during the FOT measurement, this maximal lung volume could then be referenced to TLC obtained in the body plethysmograph, which allowed absolute lung volume for each corresponding point of Grs_5_ to be indirectly determined. The absolute volumes are needed because the relationship between the changes in Grs_5_ and lung volume is non-linear and depends on absolute lung volume.

**Figure 2:**
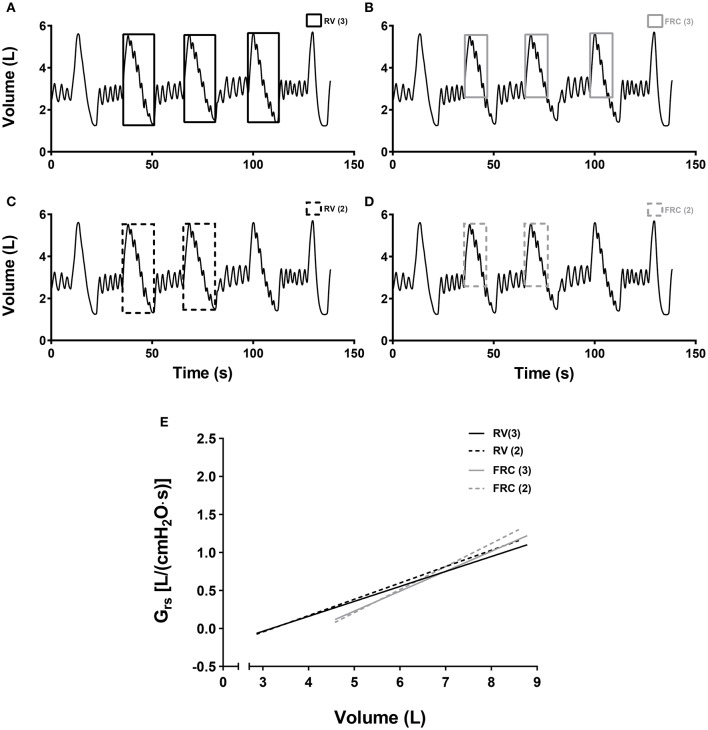
**(A–D)** Displays of the different data points taken into account to measure airway distensibility. **(A)** The squares with the solid black line enclosed the data points taken into account to measure airway distensibility when the values of the 3 deflationary maneuvers down to RV were considered (RV_3_). **(B)** The squares with the solid gray line enclosed the data points taken into account to measure airway distensibility when the values of the 3 deflationary maneuvers down to FRC were considered (FRC_3_). **(C)** The squares with the dashed black line enclosed the data points taken into account to measure airway distensibility when the values of the 2 deflationary maneuvers down to RV were considered (RV_2_). **(D)** The squares with the dashed gray line enclosed the data points taken into account to measure airway distensibility when the values of the 2 deflationary maneuvers down to FRC were considered (FRC_2_). **(E)** Linear regressions that best fitted the data of the relationship between Grs_5_ at zero flow and lung volume when the data points taking into account in **(A–D)** were included. The slopes of these lines represent airway distensibility assessed by using the data points enclosed by the squares in **A** (solid black line), **B** (solid gray line), **C** (dashed black line), and **D** (dashed gray line).

The change of Grs_5_ at zero flow and the corresponding change in lung volume represents airway distensibility (ΔGrs_5_/ΔV_L_). Airway distensibility was determined differently by using both a cubic equation and a linear equation. On one hand, the cubic equation defines a curve that best fitted the data of ΔGrs_5_/ΔV_L_ over the entire range of lung volume (Kelly et al., 2012). The curve derivatives can then be used to determine distensibility at any chosen lung volume for each visit separately. On the other hand, the linear equation traces a slope across the data points, which also defines ΔGrs_5_/ΔV_L_. Airway distensibility determined by a linear equation was calculated by pooling the data points from: 1-the three deflationary maneuvers from TLC to RV (called RV_3_); 2-the three maneuvers from TLC to FRC (called FRC_3_); 3-the first two maneuvers from TLC to RV (called RV_2_); and 4-the first two maneuvers from TLC to FRC (called FRC_2_). See Figure 2.

### Statistics

Data are shown as means ± SD. Akaike's information criterion with finite corrections (AICc) (Hurvich and Tsai, 1995) and weighted AICc (wAICc) (Wagenmakers and Farrell, 2004) were used to determine the model (linear vs. cubic) that best fitted the data of Grs_5_ at zero flow across the entire range of lung volumes (TLC to RV). In order to assess reproducibility for the values of distensibility, intra-class correlation coefficients (ICC) were calculated. Modified Bland-Altman analyses that account for repeated measurements (Bland and Altman, 1999) were then performed to test the level of agreement between the values of airway distensibility that were calculated by using data points from different ranges of lung volumes (TLC to RV vs. TLC to FRC) and for different numbers of deflationary maneuvers (all three deflationary maneuvers vs. the first two only). All statistical analyses were performed using Prism 6 (GraphPad, San Diego, CA).

## Results

**Figure 3:**
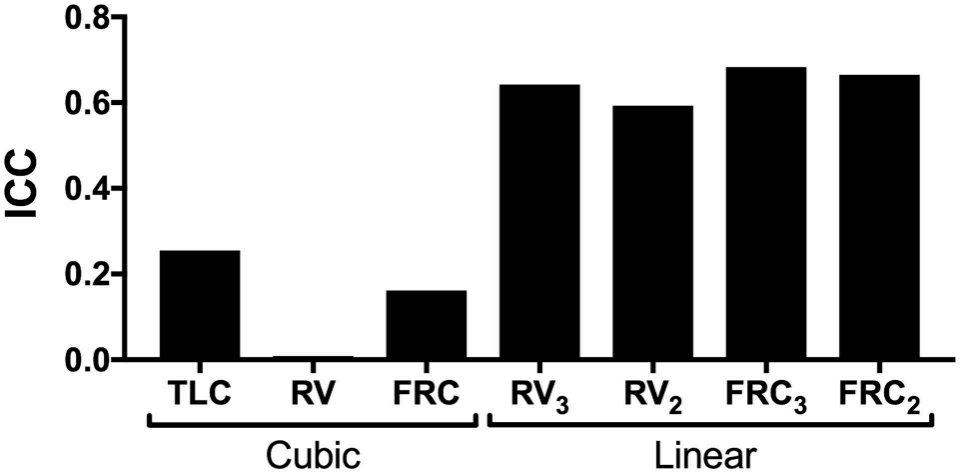
**Comparison of reproducibility between values of airway distensibility calculated using a cubic model vs. a linear model**. Airway distensibility calculated with the cubic model was determined at residual volume (RV), functional residual capacity (FRC) and total lung capacity (TLC) during all 3 deflationary maneuvers. Airway distensibility calculated with the linear model was determined across the entire range of lung volumes (RV_3_ and RV_2_) or across TLC to FRC (FRC_3_ and FRC_2_) during either all 3 (RV_3_ and FRC_3_) or only the first 2 (RV_2_ and FRC_2_) deflationary maneuvers. Each of these values of distensibility was obtained at each of the 3 visits and intra-class correlation coefficients (ICC) was used to measure reproducibility.

The demographic, lung function and the values of airway distensibility from the 13 subjects are shown in Table 1. As expected, the data points of Grs_5_ at zero flow and the corresponding lung volumes across the entire range of lung volumes better fitted a cubic curve than a linear slope (ΔAICc of −4.5 and wAICc of 0.91). However, the reproducibility for the values of airway distensibility was much superior with a linear model than a cubic model (Figure 3). Reproducibility of airway distensibility determined by a linear slope was calculated across different ranges of lung volumes and using either all three or only the first two deflationary maneuvers. When using all three deflationary maneuvers from TLC to RV (RV_3_), distensibility led to a good degree of reproducibility (ICC = 0.64). Using Grs_5_ values from all three deflation maneuvers from TLC to FRC (FRC_3_) resulted in a slightly better reproducibility (ICC = 0.68). Taking only Grs_5_ values during the first two deflationary maneuvers did worsen reproducibility when it was measured between TLC and RV (ICC for RV_2_ = 0.59) but not when it was measured between TLC and FRC (ICC for FRC_2_ = 0.66).

**Table 1:** **Subject characteristics**.

	***n* = 13**	
	**Mean**	**SD**
**DEMOGRAPHICS**		
Age (year)	26.5	4.9
Gender (male/female)	7/6	
BMI (kg/m^2^)	22.7	3.9
**SPIROMETRY**		
FEV_1_ (L)	4.1	0.8
FEV_1_ (% predicted)	102.5	10.4
FVC (L)	5.0	1.1
FEV_1_/FVC (%)	81.7	7.6
**PLETHYSMOGRAPHY**		
TLC (L)	6.4	1.5
TLC (% predicted)	103.0	10.7
FRC (L)	3.3	0.7
FRC (% predicted)	110.9	18.2
RV (L)	1.5	0.4
RV (% predicted)	101.4	19.0
**OSCILLOMETRY**		
Grs_5_ [L/(cmH_2_O·s)]	0.35	0.07
Rrs_5_ [(cmH_2_O·s)/L]	2.99	0.70
Rrs_5−19_ [(cmH_2_O·s)/L]	0.01	0.12
Xrs_5_ [(cmH_2_O·s)/L]	−0.99	0.20
A_X_ [(cmH_2_O·s)/L ·Hz]	3.01	0.95
F_res_ (Hz)	11.14	0.90
Distensibility [1/(cmH_2_O·s)]		
RV_3_	0.18	0.08
FRC_3_	0.23	0.14
RV_2_	0.19	0.08
FRC_2_	0.24	0.14

*A_X_, integrated area of low frequency reactance (i.e., when reactance is plotted as a function of frequency, A_X_ is the area enclosed by the x axis and the reactance curve from 5 Hz to F_res_); BMI, body mass index; G, respiratory system conductance; FEV_1_, forced expiratory volume in 1 s; FRC, functional residual capacity; F_res_, resonant frequency; FVC, forced vital capacity; Rrs_5_, respiratory system resistance at 5 Hz; Rrs_5–19_, Rrs at 5 Hz minus Rrs at 19 Hz; RV, residual volume; TLC, total lung capacity; Xrs_5_, respiratory system reactance at 5 Hz*.

**Figure 4:**
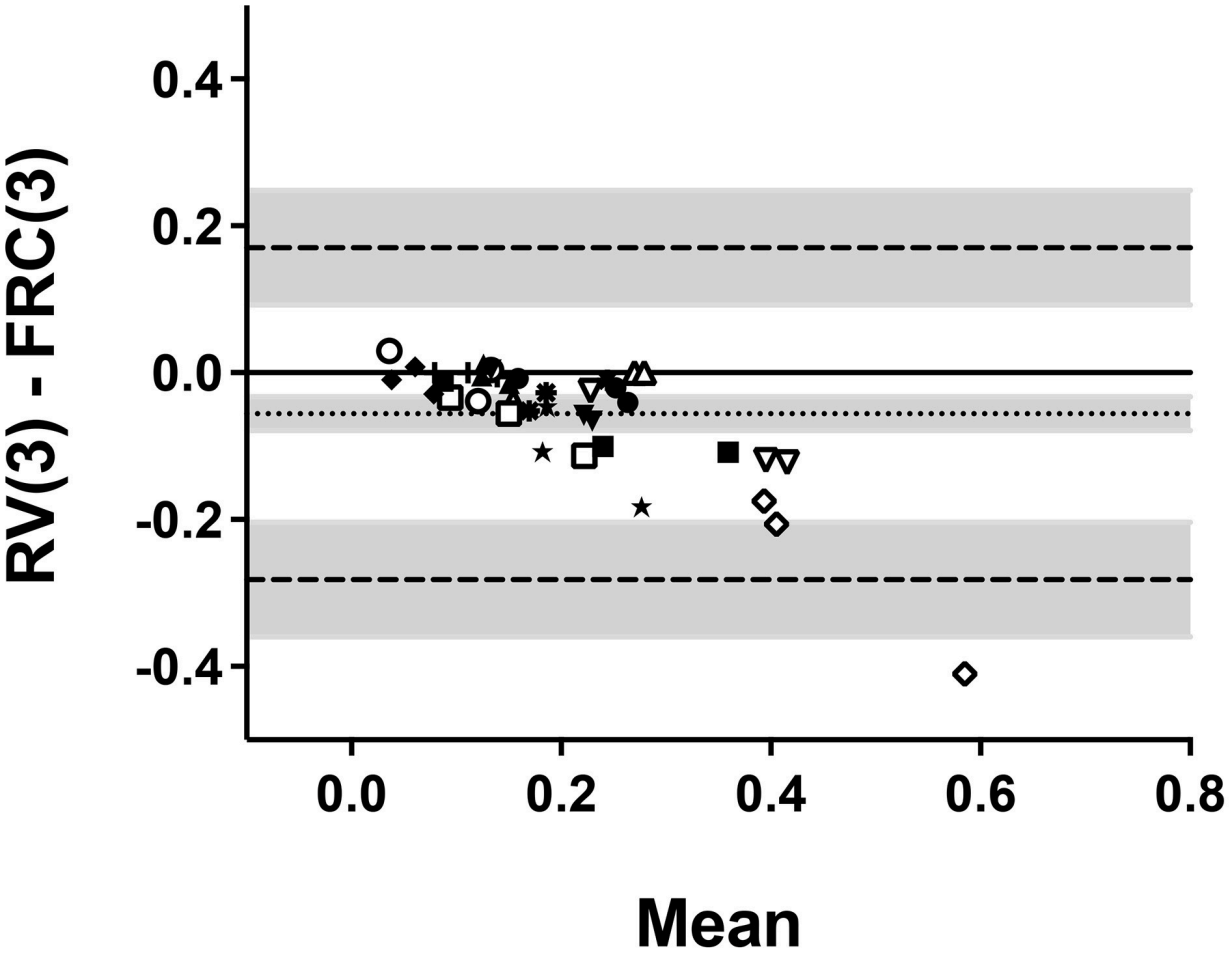
**Bland-Altman plot comparing the values of airway distensibility when all data points within the 3 deflationary maneuvers were included [RV(3)] vs. only the data points down to FRC within the 3 deflationary maneuvers were included [FRC(3)]**. Each symbol relates the difference between each value [RV(3) − FRC(3)] for each subject at each visit on the y-axis with the mean of both values [(RV(3) + FRC(3))/2] for each subject at each visit on the x-axis. Each subject is represented by a different symbol. The dotted line is the bias; i.e., the mean difference between the measurements. The dashed lines are the upper and lower limits of agreement. The shaded areas are the 95% confidence intervals for the bias and the limits of agreement.

**Figure 5:**
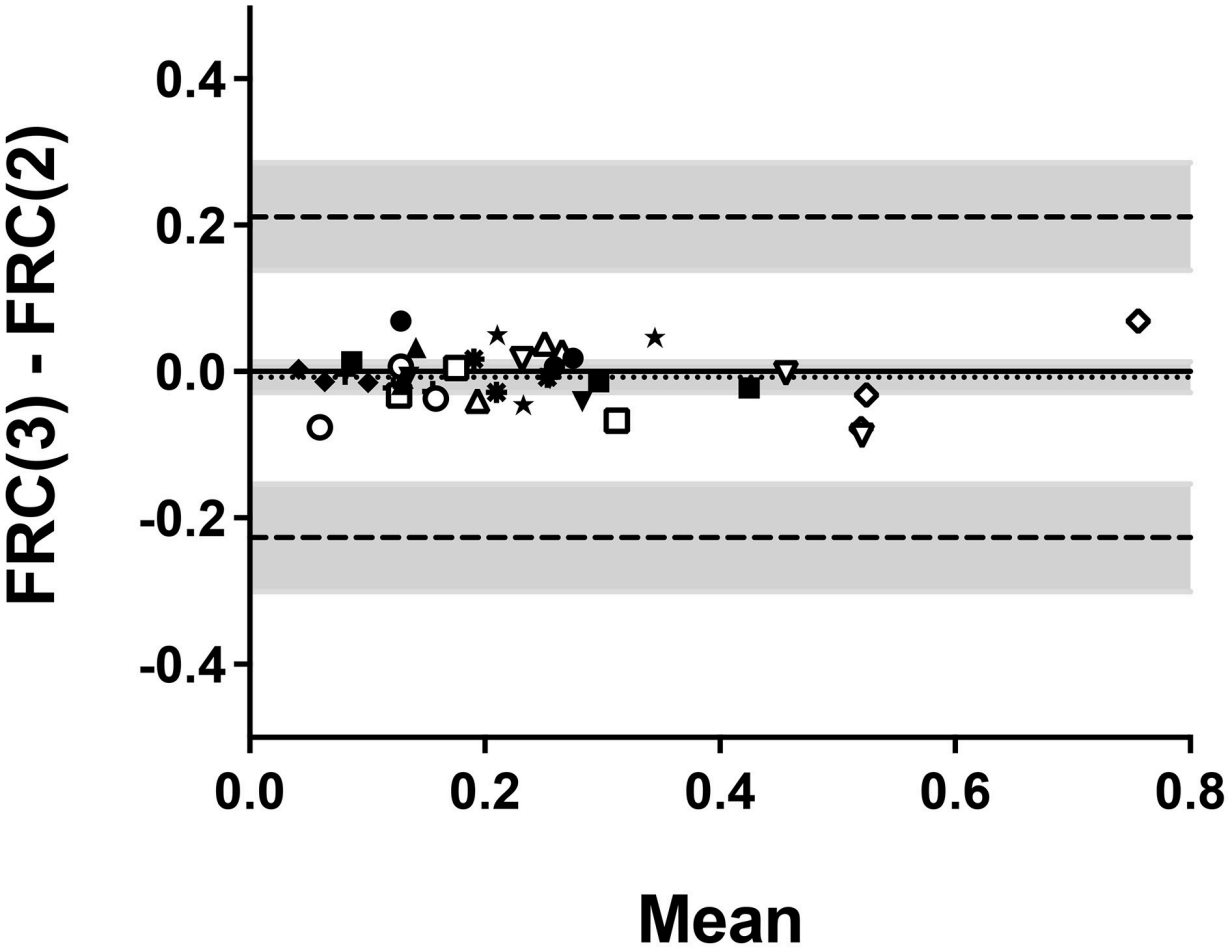
**Bland-Altman plot comparing the values of airway distensibility when only the data points down to FRC within the 3 deflationary maneuvers were included [FRC(3)] vs. only the data points down to FRC within the 2 deflationary maneuvers were included [FRC(2)]**. Each symbol relates the difference between each value [FRC(3) – FRC(2)] for each subject at each visit on the y-axis with the mean of both values [(FRC(3) + FRC(2))/2] for each subject at each visit on the x-axis. Each subject is represented by a different symbol. The dotted line is the bias; i.e., the mean difference between the measurements. The dashed lines are the upper and lower limits of agreement. The shaded areas are the 95% confidence intervals for the bias and the limits of agreement.

Bland-Altman plots were then generated to assess whether the values of airway distensibility differ depending on the range of lung volumes within which data points were taken (TLC to RV vs. TLC to FRC) and the number of deflationary maneuvers considered (two vs. three). The level of agreement between airway distensibility calculated from TLC to RV (RV_3_) vs. TLC to FRC (FRC_3_) is shown in Figure 4. The 95% confidence intervals of the mean difference between the two values do not include zero, which indicates a systematic bias. This demonstrates that, overall, airway distensibility is systematically lower when calculated for RV_3_ compared to when it was calculated for FRC_3_. The difference amounts to −0.056 ± 0.082 cm H_2_O^−1^s^−1^, representing −19.4 ± 26.7%. This negative bias also seems to be driven by higher values of distensibility. In fact, drawing a linear regression indicates that the bias increased in proportion to the value of distensibility (the slope being significantly different from zero; *p* < 0001). This was not caused by a constant coefficient of variation, as a Bland-Altman analysis using the percentage difference [RV(3) − FRC(3)/mean in %], instead of the absolute difference, on the y axis did not affect the results (the slope of the linear regression still being significantly different from zero; *p* = 0006). The level of agreement between airway distensibility calculated for FRC_3_ vs. FRC_2_ is shown in Figure 5. The mean difference between the values of airway distensibility is −0.008 ± 0.037 cmH_2_O^−1^ × s^−1^ (Table 2) and the 95% confidence intervals of the difference includes zero, which indicates no systematic bias. Thus, the values of airway distensibility measured from the first two deflationary maneuvers down to FRC are not different from those calculated from all three maneuvers down to FRC. The results of Bland-Altman analyses for all the comparisons are displayed in Table 2.

**Table 2:** **Bland-Altman statistics**.

	**RV_3_–FRC_3_**	**RV_2_–FRC_2_**	**RV_3_–RV_2_**	**FRC_3_–FRC_2_**
Bias	−0.056	−0.055	−0.009	−0.008
LoA	−0.282 to 0.170	−0.280 to 0.170	−0.148 to 0.130	−0.227 to 0.211
**95% CONFIDENCE INTERVALS**				
Bias	−0.079 to −0.033	−0.077 to −0.033	−0.022 to 0.004	−0.029 to 0.013
Upper LoA	0.092 to 0.248	0.094 to 0.246	0.084 to 0.177	0.138 to 0.285
Lower LoA	−0.360 to −0.204	−0.356 to −0.204	−0.195 to −0.102	−0.301 to −0.154

*LoA, limits of agreement; FRC, functional residual capacity; RV, residual volume*.

## Discussion

The values of airway distensibility determined using a linear model are reproducible. Our analyses also demonstrate that calculating airway distensibility with data points from TLC to FRC, instead of TLC to RV, slightly increased reproducibility. This suggests increased reliability and thus more sensitivity to detect subtle changes. Additionally, our analyses demonstrate that calculating airway distensibility from only the first two deflationary maneuvers did not affect reproducibility when the data points from FRC to RV are excluded. Thus, we suggest that two deflationary maneuvers from TLC to FRC could potentially be sufficient to measure airway distensibility by FOT, as it is a series of breathing maneuvers that would be easier to perform and should provide reproducible values.

Remodeling of the airway wall is a characteristic feature of many respiratory diseases (Hirota and Martin, 2013). The only direct way to assess airway wall remodeling in living subjects consists of harvesting specimens of the airway wall by endobronchial or open lung biopsies. Such approaches are hardly feasible for longitudinal studies because of the invasiveness of the procedures, as well as the associated expenses. Additionally, there are many technical and disease-related issues inherent to endobronchial biopsies that limit their use for the assessment of airway remodeling (Bullone et al., 2014). First, tissues can only be harvested at bronchial bifurcations, which may not represent the remodeling observed along the length of the airways. Second, bronchial biopsies only assess a few localized areas, which is problematic in patchy respiratory diseases. Finally, it cannot be performed twice at the same site, which seriously limits the studies that aim to monitor the progression or reversal of airway wall remodeling. Hence, indirect methods have been developed to assess airway remodeling. Many of them use imaging or physiological techniques to assess the degree of airway distensibility. Examples include: 1-estimations of dead space volume at different lung volumes using either N_2_ (Fowler, 1948) or CO_2_ (Carter et al., 1956) as the tracer gas; 2-the acoustic reflection technique (Hoffstein et al., 1987); 3-high resolution computed tomography (Brown et al., 2001); 4-pitot static probe (Brackel et al., 2000); and 5-anatomic optical coherence tomography (Williamson et al., 2011).

As a proof-of-concept, the degree of airway distensibility measured by the single breath nitrogen washout (SBNW) was shown to correlate with airway remodeling (Ward et al., 2001). This suggests that indirect physiological methods that assess airway distensibility are appropriate surrogates for airway wall remodeling. More importantly, this method is non-invasive and can be assessed repeatedly. SBNW also provides a measurement of the global state of the whole tracheobronchial tree, instead of representing a detailed assessment of small localized areas. However, the assessment of airway distensibility with the SBNW is time-consuming. It only provides a single estimation of dead space volume at each tested lung volume and a wash-in period of air with 100% oxygen is intercalated between each measurement. Consequently, the method is rather long and cumbersome. Two methods have been developed to assess airway distensibility within a shorter timescale, namely dead space volume using CO_2_ as the tracer gas (Johns et al., 2000) and the FOT (Brown et al., 2004). These methods are clearly useful, as they allow a relatively easy measurement of acute interventions on airway distensibility.

Forced oscillation technique (FOT) seems the most promising physiological approach to measure airway distensibility given the availability of commercial devices and the recent improvement in signal processing (Kaczka and Dellacá, 2011). FOT devices have evolved to measure the changes in respiratory system conductance (Grs) and the corresponding changes in lung volume simultaneously. When flow is zero, at end-expiration and end-inspiration, the Grs at 5 Hz mainly reflects airway caliber. The relative change in Grs_5_ over lung volume is thus a proper index of airway distensibility. The advantage over dead space volume estimations is that FOT also provides estimations of the change in airway caliber near RV and TLC. However, the method to assess airway distensibility by FOT is not standardized, has not been tested adequately for reproducibility, and the procedure may be demanding for ill subjects. Hence, there is a need to test reproducibility and to simplify the procedure to facilitate its future application in clinical research and practice.

In this study, we demonstrate that airway distensibility determined by a linear model is reproducible, irrespective of the ranges of lung volumes within which it is calculated. This is an important finding since reproducibility is essential for any test that aims to track changes over time. Our method is thus adequate to assess prospective changes of airway distensibility that occur during the course of disease development and during treatment.

The results from this study also show that the series of breathing maneuvers required to obtain a reproducible value of airway distensibility can potentially be simplified. We first recalculated airway distensibility by omitting the data points below FRC. Stopping the deflationary maneuver at FRC would greatly facilitate the assessment of airway distensibility by limiting the difficulty of breathing tidally under FRC. Conveniently, our results suggest that it could potentially also increase reproducibility. This would make the method more reliable, implying a greater sensitivity to detect small changes. Notably, the values of distensibility increased when it was calculated between TLC and FRC. We do not believe that this would compromise the measurement, but rather believe that it would improve it. This is because many confounding factors that affect Grs_5_ are highly influential at low lung volumes and of minimal influence at high lung volumes (Brown et al., 2007). These factors include tone (i.e., sustained activation of airway smooth muscle) (Kelly et al., 2012), heterogeneity in airway caliber due to varying degree of narrowing and closure/recruitment (Lutchen and Gillis, 1997; Thorpe and Bates, 1997), and the chest wall (Black et al., 2003). Stopping the maneuver at FRC would thus prevent distensibility from being affected by biased values of Grs_5_ at low lung volumes and thereby increases the validity of the measurement.

A deflationary maneuver stopping at FRC is potentially more important in disease conditions, since many of the confounding factors that affect Grs_5_ at low lung volumes are markedly enhanced in respiratory diseases (Molfino et al., 1993; King et al., 2004; Dame Carroll et al., 2015) and are thus likely to mask the actual changes in airway distensibility that occur over time. One limitation from the current study is the fact that the deflationary maneuvers were not stopped at FRC. Instead, the relevant data points from TLC to FRC were reanalyzed. The latter were obtained from the original deflationary maneuvers where the subject actually breathed from TLC to RV. Since we were not working with diseased subjects, who may perhaps be prone to recruitment difficulties after breathing at low lung volumes, we do not believe that this has affected our results in healthy subjects. However, confirmatory experiments with deflationary maneuvers actually stopping at FRC will be required to ascertain our results.

To explore whether the method can be further simplified, we recalculated airway distensibility only from the data points within the first two deflationary maneuvers. Stopping the series of breathing maneuvers after two deflationary maneuvers, instead of three, would facilitate the assessment of airway distensibility by reducing the duration of the measurement. Our results suggest that two deflationary maneuvers would be enough to provide reliable results. Together, the demonstration that airway distensibility may be obtained reproducibly without breathing below FRC and with only two deflationary maneuvers is an important finding. This simplified method would increase feasibility and thereby foster routine assessment of airway distensibility in clinical research and practice.

The fact that the degree of airway smooth muscle activation does not affect the values of distensibility at lung volumes above FRC (Brown et al., 2007; Kelly et al., 2012) is also a huge asset for the implementation of this method in clinical research and practice. It implies that airway distensibility is not affected by the actual degree of airway smooth muscle activation. Therefore, the values should not be influenced by the use of bronchodilators, such as long-acting β_2_-agonists and long-acting muscarinic antagonists. All the concerns related to withholding bronchodilators before testing can also be eliminated. Finally, it suggests that airway distensibility can be assessed either before or after short-acting β_2_-agonists without affecting the results. The method can thus be incorporated in research and clinical protocols at the most convenient time. Our simplified method to measure distensibility, which consists of two deflationary maneuvers from TLC to FRC, thus represents a perfect example of a physiological measurement that could suitably fulfill all the criteria that are required to optimize its applicability in the clinical arena. The next step will be to confirm the reproducibility of this simplified method in patients beset by respiratory diseases. The clinical applicability of this new method and its eventual implementation in clinical practice will await these confirmatory results.

This simplified method may fill an important gap in the development of a non-invasive procedure to diagnose and monitor airway remodeling. However, the extent by which airway distensibility measured with the FOT between TLC and FRC reflects remodeling remains to be ascertained. Prospective studies measuring remodeling features of the airway wall in bronchial biopsies in conjunction with measurements of airway distensibility by FOT will need to be undertaken. Parallel changes in airway remodeling and distensibility during the course of a disease or during the administration of a treatment would validate the adequacy of airway distensibility by FOT as a surrogate for airway remodeling.

## Conclusions

Airway distensibility measured by FOT is reproducible and can potentially be simplified by measuring two deflationary maneuvers from TLC to FRC. We believe that this would increase clinical usefulness by increasing reproducibility, feasibility and clinical applicability. Additionally, owing to the many confounding factors that are likely to influence Grs_5_ at low lung volumes, we believe that stopping the maneuver at FRC would increase validity. We conclude that, inasmuch as the change in airway distensibility measured between TLC and FRC reflects the changes in airway wall remodeling, this simplified method offers the possibility to monitor reliably, easily, non-invasively, and prospectively the changes in airway remodeling occurring during the natural progression of disease and during the course of a treatment.

## Author contributions

SM designed and performed some of the experiments, generated figures and contributed to manuscript writing and statistical analyses. MéL performed some of the experiments, generated figures, and contributed to statistical analyses. MB and RD contributed to manuscript writing. CH designed and performed some of the experiments. LB and MiL provided equipment and resources, and contributed to manuscript writing. GK and CF contributed to training and manuscript writing. YB provided equipment and resources for the experiments, coordinated work between centers, designed the experiments, generated the figures, and contributed to manuscript writing and statistical analyses.

## Funding

The work was supported by a donation of Merck Sharpe & Dohme Corps to the Faculty of Medicine of Université Laval, as well as the Réseau en Santé Respiratoire du FRQS (Fonds de recherche du Québec–Santé).

### Conflict of interest statement

The authors declare that the research was conducted in the absence of any commercial or financial relationships that could be construed as a potential conflict of interest.
